# Treatment outcomes among patients with drug-resistant tuberculosis managed with BPaL/BPaLM regimen in the Southeast Zone of Nigeria

**DOI:** 10.11604/pamj.2025.51.86.48418

**Published:** 2025-08-05

**Authors:** George Ikpe, Shadrach Dimang, Ufuoma Aduh, Peter Idowu Omoniyi, Ugochukwu Chukwulobelu, Charles Okafor, Emperor Ubochioma, Nneka Nwali, Godswill Nwokocha

**Affiliations:** 1 National Tuberculosis, Leprosy and Buruli Ulcer Control Program, Abuja, Nigeria,; 2 World Health Organization, Abuja, Nigeria,; 3 African Centers for Disease Control and Prevention, Abuja, Nigeria,; 4 Anambra State Tuberculosis, Leprosy and Buruli Ulcer, Control Program, Anambra, Nigeria,; 5 Imo State Tuberculosis, Leprosy and Buruli Ulcer Control Program, Imo, Nigeria,; 6 Ebonyi State Tuberculosis, Leprosy and Buruli Ulcer Control Program, Ebonyi, Nigeria,; 7 Abia State Tuberculosis, Leprosy and Buruli Ulcer Control Program, Abia, Nigeria

**Keywords:** Treatment outcomes, multidrug-resistant tuberculosis, BPaLM regimen

## Abstract

**Introduction:**

the treatment of multidrug-resistant tuberculosis (MDR-TB) has been challenging, often associated with life-threatening adverse events and poor treatment outcomes. The recent World Health Organization (WHO) recommendation of the BPaLM regimen, consisting of bedaquiline, pretomanid, linezolid, and moxifloxacin for treatment of pre-extensively drug-resistant tuberculosis (Pre-XDR) and rifampicin-resistant tuberculosis (MDR-TB/RR), has been shown to have favorable treatment outcomes. This study focused on the outcomes of patients managed with this regimen in the southeastern zone of Nigeria between November 2023 and October 2024, following approval by the National Tuberculosis and Leprosy Control Program (NTBLCP) in November 2023.

**Methods:**

patient demographic and clinical data were collected from DR-TB treatment registers, active drug safety monitoring forms (aDSM), and laboratory registers using an abstraction tool. Data were analyzed using descriptive statistics such as frequency and percentages for variables such as age, gender, adverse events, new and retreatment cases, treatment outcomes such as completed treatment, cured, loss to follow-up (LTFU), and death.

**Results:**

out of the 212 patients enrolled for treatment during the period, 202 patients were treated for MDR-TB/RR with BPaLM, while 10 were treated for Pre-XDR with BPaL. The TSR for the MDR-TB/RR category is 89%, while the TSR for the Pre-XDR category is 100%. More than 6.7% of the patients in the MDR-TB/RR category were LFTU, and another 6.7% died. Over 86% of the patients treated had no documentation for adverse events, while 54% of the MDR-TB/RR cases were new cases.

**Conclusion:**

using the BPaLM regimen for DR-TB treatment results in favorable patient outcomes.

## Introduction

The emergence of MDR-TB is posing a substantial challenge to achieving the desired goal of ending the epidemic of tuberculosis (TB) by 2030, because it is more difficult and expensive to treat than drug-sensitive TB. Furthermore, culture, regarded as the gold standard for diagnosing and monitoring patients, takes a long time, up to 4-8 weeks, for the release of results. Current molecular tests, such as GeneXpert and TrueNat, are limited in their scope, providing only a limited view of the resistance profile of both first-line and second-line drugs, thereby making drug selection somewhat challenging. Estimates show that up to half a million new cases of MDR-TB are diagnosed yearly, with over 150,000 deaths globally [1]. Nigeria is ranked 6^th^ among the 30 “high burden TB countries" which constitute over 80% of the world´s cases, and among the 14 countries heavily burdened by Multidrug Resistant Tuberculosis (MDR-TB) [2].

The actual picture of MDR-TB in the African continent and the country is somewhat lacking. There is a paucity of data about the true burden of MDR-TB in Nigeria, as the country is just rounding off with its 2^nd^ Drug Resistant TB Survey (DRS), the last one was in 2012. Drug-resistant TB surveillance has not been routine as the WHO recommends due to funding constraints. However, modelled estimates revealed that there are over 90,000 cases of MDR-TB, with almost half of these occurring in Nigeria and South Africa, and almost two-thirds of these cases are not reported [3]. This same study also found that deaths among patients with MDR-TB were a little above 20% and were almost half among those with extensively drug-resistant tuberculosis (XDR-TB). In Nigeria, current estimates of MDR-TB among new TB cases are 1.6% and among previously treated or retreatment TB cases are 9.8% according to the global TB report of 2024 [4].

The National Tuberculosis and Leprosy Control Programme (NTBLCP) commenced the clinical and programmatic management of Drug-Resistant Tuberculosis (PMDT) in 2010 with the enrolment of 25 patients at the University College Hospital in Ibadan. This followed the World Health Organization (WHO) recommendation on the “STOP TB strategy” to address issues of MDR-TB treatment globally [5] and the conduct of the first National MDR-TB prevalence survey. By 2016, the WHO recommended the use of the shorter MDR-TB treatment regimen with the use of injectables, which was well accepted due to the shorter duration of treatment [6]. The all-oral shorter treatment regimen was adopted by NTBLCP in 2018 with scale up across the country in 2019 and by 2020, the modified all oral shorter treatment regimen comprising of BPaL (bedequilline, pretomanid, linezolid) operational research commenced in 4 states in Nigeria, namely, Lagos, Kano, Bauchi and Adamawa states for the management of Pre-XDR-TB disease due to mycobacterium complex resistant to rifampicin and also any of the fluoroquinolones such as levofloxacin or moxifloxacin [7]. Consequently, the preliminary report of the pilot implementation of the BPaL regimen showed a treatment success (TSR) of 91.2% which was higher than the national TSR of 83% at the same period in 2022. This regimen, according to the pilot study, showed improved efficacy and high tolerability, and better treatment outcomes compared to previous treatment options that were associated with lower treatment success rates and adverse events.

In addition, the WHO in 2022 updated recommendation for the use of the novel BPaL regimen for 6-9 months under programmatic conditions, including the BPaLM regimen for 6 months in the treatment of MDR-TB/RR and Pre-XDR patients [2]. The NTBLCP, based on the WHO recommendations and experiences from the pilot implementation, the TB PRATECAL study, and the ZeNix trial [8,9], recommended the use of BPaL(M) regimen under programmatic conditions nationwide from November 2023 [10]. This recommendation was for the treatment of MDR-TB/RR and Pre-XDR TB for patients aged 14 years and above. This directive was hinged on the phasing out of the use of second-line injectable drugs for the management of MDR-TB in 2020, following global consensus [11].

Shorter treatment regimens have been shown to lead to better treatment outcomes in terms of TSR compared to longer treatment regimens used previously [12]. Studies conducted all over the world have found similar high treatment success rates of above 90% associated with the use of the BPaL(M) regimen, with a reduction in incidence of adverse effects, demonstrating the efficacy and tolerability of the regimen [13,14]. Globally, it is reported that the TSR for MDR-TB has improved from 50% in 2012 to 60% in 2019 [15]. Nevertheless, some of the drugs in the regimen were found to be associated with adverse events that may resolve with a reduction in dosage or discontinuation. Peripheral neuropathy, optic neuropathy, and myelosuppression were found to be associated with linezolid at a dose of 1200mg, while prolongation of the QT interval and hepatotoxicity were found to be associated with bedaquiline [1,9].

In a 24-week trial study to evaluate the efficacy and safety of the regimen with linezolid at 600mg once daily, over 20% of the patients experienced peripheral neuropathy, and more than 7% experienced optic neuritis, which resolved after some time after completion of treatment [9]. However, the commonest side effects associated with this regimen, as reported, were gastrointestinal side effects, which include nausea and vomiting, and sometimes dizziness, and resolve with ancillary medications [16].

### Rationale

Following recommendations by the WHO in 2022 [2] and experiences from BPaL pilot implementation, the NTBLCP approved the scaling-up of the BPaL(M) regimen nationwide under programmatic conditions starting from 1^st^ November 2023. However, there is no information regarding the outcomes of treatment of patients managed with this regimen in the southeast zone of Nigeria, including their adverse events under programmatic settings, being a novel regimen following the nationwide recommendation by the NTBLCP. To our knowledge, this is the first study in the country outside the pilot study, which involved only 4 states, that evaluated the treatment outcomes of patients managed with this regimen. We hope that the results and recommendations will add to the growing body of knowledge about the treatment outcomes with the regimen. The information from this study will be very beneficial in shaping treatment policy in the National TB program with regard to the new regimen and lead to improved program implementation and treatment outcomes for our patients.

### Objectives

1) To identify the outcomes of treatment of patients diagnosed as having MDR-TB/RR and Pre-XDR TB and managed with BPaLM and BPaL, respectively, between 1^st^ November 2023 and 31^st^ October 2024 in the Southeastern zone of Nigeria, and these outcomes include cure rates, treatment completed, loss to follow-up and death for the same period. 2) To determine the key adverse events associated with the use of the drug BPaL(M). 3) To determine what proportion of the cases are new cases and retreatment cases. 4) To determine what proportion of the cases are co-infected with HIV.

## Methods

**Study setting:** the study was carried out in the Southeast zone of Nigeria, consisting of 5 states, namely: Abia, Anambra, Ebonyi, Enugu, and Imo States. Nigeria has a population of over 220 million by projection, according to the 2006 population census. It is divided into 6 Geopolitical zones, which are the Northwest, Northeast, North-Central, Southwest, Southeast, and South-South Zones respectively. The Southeast zone has an estimated population of 29 million residents according 2006 population census by projection estimation [17]. The governance of the country and the zones is premised on three tiers of which are the Federal, State, and Local governments. The health structure is also distributed on the same premises, which are the Tertiary facilities owned by the Federal Government, the secondary facilities supported by the state Ministry of Health, and the Primary Health Care services, which are coordinated by the Local Government Areas. DR-TB treatment in the country has been decentralized in the country including the zones, to the Local Government Areas, where patients are managed on an outpatient basis, except those who are critically ill, who are managed at the treatment centers in the states.

**Study population:** the study was carried out among patients diagnosed with MDR-TB and Pre-XDR TB who were managed between November 2023 and October 2024 with the BPaL(M) regimen, respectively, and are 14 years old and above according to the standard operating procedure of NTBLCP [11].

**Study design:** this was a cross-sectional descriptive study that focused on all the DR-TB patients enrolled for treatment from November 1^st^, 2023, to October 31^st^, 2024, in the southeast zone and managed using the BPaL (M) regimen.

**Data collection:** patient data were abstracted from DR-TB treatment registers, active drug safety and monitoring (aDSM) forms, and GeneXpert laboratory registers using a standardised data abstraction tool. Data collected include age, sex, adverse events, new and retreatment cases, treatment outcomes such as cured, treatment completed, loss to follow-up, and death.

**Data analysis:** the data were summarized using descriptive statistics such as the frequency and percentages, as seen in the tables under the results section below. The variables used in the analysis were age group, gender, adverse drug events, treatment type, such as new and retreatment cases, treatment outcomes, such as cured, treatment completed, loss to follow-up, and death. The cure rate was calculated as the total number of bacteriologically positive cases with negative culture result at the end of treatment divided by the total number of bacteriologically positive cases diagnosed with by either GeneXpert or TrueNat (WHO-approved molecular tests) multiplied by 100. The treatment success rate was calculated as the total number of cases declared cured with evidence of a negative sputum culture result at the end of treatment plus the total number of cases declared treatment completed due to absence of sputum culture result at the end of treatment divided by the total number of cases enrolled within that period, multiplied by 100.

**Ethical approval:** the ethical approval for this study was sought and obtained from the Abia State Ministry of Health with approval number ASMH/EC/25/011.

## Results

A total of 212 patients were bacteriologically diagnosed, enrolled, and treated for MDR-TB/RR and Pre-XDR TB in the southeast zone of Nigeria between November 1^st^, 2023, and October 31^st^, 2024, following NTBLCP nationwide scale-up for DR-TB treatment using BPaL(M) regimen following the dosage on the country´s SOP (Table 1). Our data revealed that out of the 212 patients who were enrolled for DR-TB treatment, 10 patients were diagnosed as having Pre-XDR and treated with BPaL regimen, while 202 patients were diagnosed as having MDR-RR and treated with BPaLM regimen. The patients were 15 years and above, as the SOP recommended that the regimen is not for children 14 years and below. The age and sex distribution of patients diagnosed and enrolled for DR-TB with the BPaL/BPaLM regimen is presented in Table 2. The findings indicate that the majority, 158 (74.5%) of the total patients who were enrolled were females. It also revealed that more than 117 (55%) of the patients were above 40 years. Findings from Table 2 also revealed that 6 (2.8%) of the patients enrolled during the period under study were found to be co-infected with HIV.

**Table 1:** recommended dosing for the BPaL(M) regimen

Drug	Dosage
Bedaquiline (100 mg tablet)	400mg once daily for 2 weeks, then 200mg 3 times a week
Pretomanid (200mg tablet)	200mg once daily
Linezolid (600mg tablet)	600mg once daily
Moxifloxacin (400mg tablet)	400mg once daily

Source: standard operating procedure for BPaL(M) scale-up for program staff and health care providers, (NTBLCP) version 1, October 2023

**Table 2:** age, sex, and HIV background of enrolled DR-TB patients in the Southeast Zone of Nigeria

Characteristics	Frequency; N =212	Percentage
**Sex**		
Male	54	25.5
Female	158	74.5
**Age group**		
15 - 19	9	4.2
20 - 24	10	4.7
25 - 29	20	10.7
30 - 34	27	12.7
35 - 39	29	13.7
40 and above	117	55.2
HIV co-infection	6	2.8

Up to 86% of the total patients who were enrolled had no documentation for adverse events, and therefore, we could not perform further analysis on the data. For the BPaL treatment category, 9 (90%) of the patients treated with this regimen were reported as cured, and only 1 (10%) of the patients treated with this regimen was reported as having completed treatment, giving a treatment success rate of 100%. For the other outcomes, such as loss to follow-up and death, they were reported as zero. For the BPaLM treatment category, 88 (55.3%) patients were found to be cured, and 55 (33.3%) were found to have completed treatment, thereby giving a treatment success rate of 89%. However, 11 (6.7%) patients died, and another 11 (6.7%) were reported to be lost to follow-up (Table 3). For the type of DR-TB case diagnosed, only one DR-TB case was diagnosed as a new case for the Pre-XDR category, while the majority, 6(85.7%), were new cases. For the MDR-RR treatment category, 74 (58.7%) were diagnosed as new cases, while 52 (41.3%) were diagnosed as retreatment cases (Table 4).

**Table 3:** treatment outcomes of DR-TB patients managed with BPaL(M) in the Southeast Zone of Nigeria

Treatment type	Pre XDR	RR/MDR
New case	1 (14.3)	74 (58.7)
Retreatment	6 (85.7)	52 (41.3)

** missing data =77; Pre-XDR: pre-extensive drug-resistant tuberculosis; RR/MDR: rifampicin-resistant/multidrug-resistant tuberculosis; DR-TB: drug-resistant tuberculosis

**Table 4:** type of DR-TB cases diagnosed in the Southeast Zone

Treatment type	Pre XDR	RR/MDR
New case	1 (14.3)	74 (58.7)
Retreatment	6 (85.7)	52 (41.3)

Pre-XDR: pre-extensive drug-resistant tuberculosis; RR/MDR: rifampicin-resistant/multidrug-resistant tuberculosis; DR-TB: drug-resistant tuberculosis

## Discussion

The findings from this study show that females constituted almost three-quarters of the patients enrolled for DR-TB treatment within the period, compared to men who made up about a quarter of all the patients. Although studies have shown that TB affects more men than women, women, on the other hand, have been shown to have better health-seeking behavior than men and therefore are more likely to seek health care than men [18]. The majority of patients enrolled were in the age category of over 40 years, in agreement with other studies that found that the risk of development of tuberculosis increases with age because of a decline in the immune system, and the risk of other co-morbidities such as diabetes mellitus is common among those in the older population [19]. However, there was no recorded data about other co-morbidities except HIV co-infection, which was 6 (2.8%) among those enrolled for DR-TB treatment during the period, despite the SOP checklist, which recommends baseline investigations for patient enrollment, which includes fasting blood sugar for diabetes mellitus [10].

Programmatically, DR-TB cases are divided into new cases and retreatment cases. New cases are patients that have never received TB medications or had received medications for less than 4 weeks, and retreatment cases or previously treated cases, patients who have received TB medications for more than 4 weeks or more [20]. The majority of patients diagnosed under the Pre-XDR category were retreatment or previously treated cases and this is not surprising, as similar findings were observed by Nwachukwu *et al*. in Southeastern Nigeria in 2023, in which all patients in the retreatment category of DR-TB were Pre-XDR [21]. Nevertheless, a greater proportion of patients in the MDR-RR category were new cases, which is a very big concern for the program, because it is an indicator for ongoing community transmission [22]. However, some other authors are of the opinion that some of those cases termed as new may be patients in the previous exposure that was not properly classified [23].

The cure rates for the BPaL category within the period, which are above 90% and the treatment success rate of 100% further reinforce evidence from the pilot implementation in the 4 states of the country and other studies, such as the ZeNix trial about the efficacy of the regimen over 6 months [9]. There was no record of deaths, loss to follow-up and treatment failure. For the BPaLM category, the TSR is 89% which is a finding similar to most studies on the outcomes of treatment with the regimen [14]. A shorter regimen for 6 months has been shown to improve compliance, reduce the number of adverse events, and loss to follow-up [13]. It is believed that the shorter duration and reduced number of pills associated with this regimen will significantly help to improve the strain on the health system due to DR-TB. However, 11 (6.7%) patients were found to have died, and another 11 (6.7%) were lost to follow-up in the BPaLM treatment category. It could not be ascertained from the study the cause of deaths of these patients and the reason for the loss to follow-up, or whether they were pre-treatment loss to follow-up. Nevertheless, decentralization of treatment by NTBLCP has helped reduce loss to follow-up among patients on treatment for DR-TB in the country.

We could not report findings for the key adverse events as set out in the objectives because of poor documentation by health care workers monitoring the treatment of the patients. However, the documented key adverse event that was observed for the BPaL category was ototoxicity, but we cannot ascertain whether this was an overlapping toxicity with other medications, and there was no documentation to show if the patient was taking any other medication. Apart from the aminoglycosides, which have been established to cause ototoxicity, little is known about the adverse events with this regimen [24].

Moreover, for the BPaLM category, the key adverse events documented were optic neuritis and peripheral neuropathy, jaundice, and arrhythmias. Hepatotoxicity and arrhythmias have been found to be associated with bedaquiline, which has been shown to produce a cytotoxic metabolite in the liver during its metabolism by the cytochrome P450 enzyme system and elongation of the QT interval, respectively [25]. Optic neuritis and peripheral neuropathy are associated with linezolid, which has also been known to cause myelosuppression [14,25]. We believe that the key adverse events may have been more than the data we have, but we presume that there was poor monitoring of patients as well as poor documentation by the health workers in the different states due to a lack of capacity.

## Conclusion

Based on the WHO recommendation for the use of BPaL and BPaLM for the treatment of Pre-XDR TB and MDR-RR TB, respectively, in 2022 and the NTBLCP directive for nationwide scale up after a pilot study in 4 states in the country in 2023, this study focused on the treatment outcomes of patients managed with this regimen in the Southeastern zone of Nigeria and found high treatment success rates above 90% which is comparable to other rates found in other studies across the globe and the pilot study. Nonetheless, this study only looked at the outcomes regarding the regimen and could not study the relationships to determine the association between the regimen and some of the outcomes. We did not follow the patients to determine post-treatment outcomes. There is a lot of missing data, such as on the adverse events and treatment outcomes, which were not documented, and we acknowledge this as a limitation of the study.

### What is known about this topic

- The use of this regimen BPaL(M) for DR-TB treatment with a shorter duration gives a high treatment success rate compared to previous DR-TB regimens that are longer.

### What this study adds

- High treatment success rates associated with the use of this regimen for DR-TB treatment of over 90%;
- The increasing number of new cases of MDR-RR TB in the community of over 58% underscores the need for improvement of DR-TB surveillance measures;
- There is sub-optimal monitoring of DR-TB patients on treatment with this regimen, evidenced by non-reporting using the active drug safety monitoring forms; therefore, there is a need to build the capacity of health care workers on DR-TB patient monitoring.
